# Improved accuracy of co-morbidity coding over time after the introduction of ICD-10 administrative data

**DOI:** 10.1186/1472-6963-11-194

**Published:** 2011-08-18

**Authors:** Jean-Marie Januel, Jean-Christophe Luthi, Hude Quan, François Borst, Patrick Taffé, William A Ghali, Bernard Burnand

**Affiliations:** 1 Institute of Social and Preventive Medicine, CHUV and University of Lausanne, Switzerland; 2 Health Observatory, Canton of Valais, Switzerland; 3 University Hospital of Geneva, Switzerland; 4 Center for Health and Policy Studies, University of Calgary, Calgary, Alberta, Canada; 5 Department of Community Health Sciences, University of Calgary, Alberta, Canada; 6 Department of Medicine, University of Calgary, Alberta, Canada

**Keywords:** ICD-10, Agreement, Administrative Data, Co-morbidity

## Abstract

**Background:**

Co-morbidity information derived from administrative data needs to be validated to allow its regular use. We assessed evolution in the accuracy of coding for Charlson and Elixhauser co-morbidities at three time points over a 5-year period, following the introduction of the International Classification of Diseases, 10th Revision (ICD-10), coding of hospital discharges.

**Methods:**

Cross-sectional time trend evaluation study of coding accuracy using hospital chart data of 3'499 randomly selected patients who were discharged in 1999, 2001 and 2003, from two teaching and one non-teaching hospital in Switzerland. We measured sensitivity, positive predictive and Kappa values for agreement between administrative data coded with ICD-10 and chart data as the 'reference standard' for recording 36 co-morbidities.

**Results:**

For the 17 the Charlson co-morbidities, the sensitivity - median (min-max) - was 36.5% (17.4-64.1) in 1999, 42.5% (22.2-64.6) in 2001 and 42.8% (8.4-75.6) in 2003. For the 29 Elixhauser co-morbidities, the sensitivity was 34.2% (1.9-64.1) in 1999, 38.6% (10.5-66.5) in 2001 and 41.6% (5.1-76.5) in 2003. Between 1999 and 2003, sensitivity estimates increased for 30 co-morbidities and decreased for 6 co-morbidities. The increase in sensitivities was statistically significant for six conditions and the decrease significant for one. Kappa values were increased for 29 co-morbidities and decreased for seven.

**Conclusions:**

Accuracy of administrative data in recording clinical conditions improved slightly between 1999 and 2003. These findings are of relevance to all jurisdictions introducing new coding systems, because they demonstrate a phenomenon of improved administrative data accuracy that may relate to a coding 'learning curve' with the new coding system.

## Background

Administrative data are widely used to examine various health and policy related issues, such as health outcomes, utilisation of services, quality of care and surveillance [1-6]. One major asset of using administrative data is that they cover large populations, are ready to be analyzed, and contain detailed clinical and outcome information. There have been a number of studies assessing the accuracy of administrative data [7-16]. However, the validity of using administrative data for research purposes has been questioned repetitively [7-10] because several studies demonstrate variable and sometimes suboptimal agreement between chart data and administrative data [11-16].

Studies have shown variable accuracy across jurisdictions. Relatively little is known about trends over time in accuracy of coding and, in particular, coding "learning curves" after introduction of a new coding system. Canada, for example, introduced the International Classification of Diseases, 10^th ^revision, (ICD-10) [17] recently, between 2002 and 2007, [18] with each province doing so on a different schedule, and there was no available information on the possible existence of coding learning curve effects on data validity. One study assessed the accuracy one year after the introduction in Alberta (Canada), [19] but without time trend investigation. ICD-10 was introduced in Switzerland in 1998. This study assesses data accuracy in the five years following the implementation of ICD-10 in the French part of Switzerland, with assessments of administrative data accuracy relative to charts in 1999, 2001, and 2003. Our primary research question of interest was to determine whether there was evidence of improved data accuracy.

## Methods

### Study Population

This time trend evaluation study was based on three cross-sectional analyses of randomly selected administrative discharge records from three Swiss hospitals collected in 1999, 2001 and 2003. Two teaching hospitals and one non-teaching were included. Each of the two teaching hospitals has more than 1000 beds and over 30'000 discharges per year, while the non-teaching hospital has 300 beds and 12'000 discharges per year. For these three hospitals, the data were used for reimbursement at the date of the study start. Professional coders were nurses with at least 5 years of clinical practice and at least one year of training as a coder. Professional coders were introduced before 1999 for one teaching hospital and between 1999 and 2001 for the one other teaching and the non-teaching hospital. We randomly selected 500 records among patient discharges in 1999, 2001 and 2003 from each hospital, thus collecting 1500 records from each hospital, totalling 4'500 records. We included patients 16 years of age or older who stayed in the hospital for at least 24 hours and were discharged from any acute care wards from these hospitals. Of the 4'500 randomly selected patients, we excluded 1001 patients for the following reasons: eight patients because they left the hospital against medical advice, three because their age was less than 16 years, 11 because their length of stay (LOS) was less than 24 hours, and 979 because their charts could not be located.

The study was approved by the respective ethic committees of the three cantons.

### Chart Data Abstraction

We identified charts through the chart number recorded in the administrative data. Chart abstraction was performed by two trained research nurses who read the entire chart, including admission and transfer notes, physician daily progress notes and orders, consultation notes, operative notes, diagnostic imaging and exam reports, discharge and narrative summaries and pathology reports. One research nurse undertook the data abstraction for each chart. The detailed chart review process took approximately 45 minutes per chart, on average. The reviewers extracted information about patient age and sex, length of stay, death and Charlson [20] and Elixhauser [21] co-morbidities. Presence or absence of these co-morbidities was determined using the definitions described by Charlson et al. [20] and a chart abstraction instrument developed by a Canadian research team [19] for determining the Elixhauser co-morbidities [21]. All diagnoses corresponding to the definition of each non-redundant co-morbidity from Charlson and Elixhauser indices were identified by the two research nurses in the medical records. We excluded the condition 'Other neurological disorders' from the chart review process because its definition is too broad and vague. The inter-rater agreement between the two reviewers was assessed before the chart abstraction process. Kappa values ranged from -0.04 to 1.00 among 50 charts. Out of 30 co-morbidities assessed, 16 had substantial agreement (Kappa: 0.60-0.79), 10 had moderate agreement (Kappa: 0.40-0.59), and 4 had fair or poor agreement (Kappa< 0.40) [22]. We further trained the reviewers through discussion of results from this agreement test. Chart data abstraction was performed between 2005 and 2007.

### Defining Co-morbidities in Administrative Data

Charlson [20] and Elixhauser [21] indices were derived from ICD10 coding algorithms. Each administrative hospital discharge record contains a unique personal identification number, patient chart number, and up to 30 diagnoses in one teaching hospital, and up to 10 diagnoses in the other teaching hospital and in the non-teaching hospital. We used a recently developed ICD-10 coding algorithm [23] to define the 36 non-redundant Charlson and Elixhauser co-morbidities in these administrative data.

### Matching comparisons between ICD-10 data and Chart Review Data

Comparisons were based on the match between ICD-10 data and chart review data for the same hospital discharges, in the three hospitals samples and studied years, respectively, using the unique personal identification number. As explained above, we developed two comparative databases, one using ICD-10 data and the other using chart review. For each hospital discharge, we matched chart review data and non-redundant co-morbidities from Charlson and Elixhauser indices that had been identified using ICD-10. Then, we performed pairwise analyses that were constituted by ICD-10 data and chart review data for all hospital discharges in our study and for all non-redundant Charlson and Elixhauser indices co-morbidity, respectively.

### Statistical Analysis

The prevalence of the Charlson and Elixhauser co-morbidities in administrative data and chart review, as well as their difference (i.e. prevalence difference, Δ*_Chart-ICD_*), were assessed for each year and co-morbidity. Ninety-five percent confidence intervals for Δ*_Chart-ICD _*were calculated using the formula for the comparison of two binomial proportions, accounting for sampling weights. The heterogeneity in prevalence differences across the three years was assessed by the Cochran *Q *statistic for each co-morbidity, respectively [24].

The accuracy of the administrative data was determined using chart data as the 'reference standard'. We calculated the sensitivity (Se) and positive predictive value (PPV) for each co-morbidity and year. We assessed the impact of the year using logistic regression analysis for survey sampling and global Wald test. The dependent variable was the "sensitivity" and the study year was a covariate (the reference year was 1999 and an odds-ratio was estimated for 2001 and 2003, respectively). A P-value < 0.05 was considered as significant.

Using a different perspective, we also calculated Cohen's Kappa index value along with its 95% confidence interval to assess the agreement between the ICD-10 and Chart review for each co-morbidity and year. These 95% CI intervals were used to assess pairwise differences between the indexes [25,26]. No multiple testing adjustments were performed [27]. Finally, the co-morbidity counts, determined for both Charlson and Elixhauser items, for the ICD-10 and chart review data were grouped into 4 ordinal categories. The categories were 0, 1, 2 or 3, and 4 or higher for the Charlson index, and 0, 1 and 2, 3-5, and 6 and higher for the Elixhauser index. A weighted Kappa index between ICD-10 and chart review data, with weights 1-|i-j|/(4-1) where i and j indicates the rows and columns of the 4 categories, respectively, was calculated for each year.

Various sampling fractions were used, as the number of missing or excluded patients differed across hospitals and years: 0.962 (481/500) for the first teaching hospital, 0.722 (361/500) for the second teaching hospital and 0.632 (316/500) for the non-teaching hospital in 1999, 0.952 (476/500) for first teaching hospital, 0.806 (403/500) for second teaching hospital and 0.632 (316/500) for the non-teaching hospital in 2001, and 0.992 (496/500) for first teaching hospital, 0.910 (455/500) for the second teaching hospital and 0.390 (195/500) for the non-teaching hospital in 2003.

All statistical analyses were performed using the SAS Software version 9.2, Cary, NC, USA.

## Results

We analyzed 3'499 patient records (77.8% of 4500). The mean age (standard deviation) was 58.1 years (19.2) in 1999, 57.3 (20.5) in 2001, and 55.3 (20.1) in 2003 (p = 0.42). The sex ratio (Female/Male) was 1.14 in 1999, 1.08 in 2001, and 1.05 in 2003 (p = 0.58).

The mean number of diagnoses coded was 4.01 (2.8), across all hospitals. The mean number of diagnoses differed between the three hospitals (p < 0.001): 4.99 (2.8) in the first teaching hospital with up to 30 diagnoses coded, 3.29 (2.7) in the second teaching hospital with up to 10 diagnoses coded, and 3.33 (2.4) in the non teaching hospital with up to 10 diagnoses coded. Table 1 presents the prevalence of the 36 co-morbidities by data source across study years. In general, administrative data underreported 33 conditions but reported similar levels of moderate and severe liver disease, any tumour and lymphoma compared to chart review data. The prevalence difference, Δ*_Chart-ICD_*, ranged from -0.5% to 15.1% in 1999, from -0.6% to 16.1% in 2001, and from -1.7% to 12.2% in 2003. The difference in trend Δ*_Chart-ICD _*across the three years was significant for 33 co-morbidities. No significant differences were observed for myocardial infarction, any tumor and metastatic cancer.

**Table 1:** Prevalence of Charlson and Elixhauser Co-morbidities in ICD-10 Administrative and Chart Data by Study Year, N = 3'449*

	Prevalence of co-morbidities						Prevalence difference, Δ*_Chart-ICD_*						
	
	1999 (n = 1'158)		2001 (n = 1'195)		2003 (n = 1'146)		1999		2001		2003		
	
COMORBIDITIES	ICD-10	Chart	ICD-10	Chart	ICD-10	Chart	Δ	(95%CI)	Δ	(95%CI)	Δ	(95%CI)	P-value**
	N1	N2	N1	N2	N1	N2	*%*	*%*	*%*	*%*	*%*	*%*	
Cardiac arrhythmias^2^	85 (5.7)	237 (15.9)	133 (8.9)	292 (19.5)	144 (9.6)	229 (15.3)	10.2	(9.1-11.3)	10.6	(9.3-11.9)	5.6	(4.0-7.3)	< 0.001
Congestive heart failure^1,2^	76 (5.1)	302 (20.2)	80 (5.3)	285 (19.0)	85 (5.6)	268 (17.9)	15.1	(13.8-16.4)	13.7	(12.5-14.1)	12.2	(10.5-14.0)	< 0.001
Myocardial infraction^1(a)^	49 (3.2)	64 (4.3)	57 (3.8)	48 (3.2)	57 (3.8)	70 (4.7)	1	(0.3-1.8)	-0.6	(-1.3-0.1)	0.9	(-0.4-2.2)	0.437
PVD^1,2^	43 (2.9)	159 (10.6)	74 (4.9)	316 (21.0)	59 (3.9)	168 (11.2)	7.7	(6.9-8.5)	16.1	(14.9-17.3)	7.3	(6.1-8.4)	< 0.001
PCD^2^	20 (1.4)	49 (3.3)	31 (2.1)	68 (4.5)	27 (1.8)	55 (3.7)	1.9	(1.4-2.5)	2.5	(1.8-3.1)	1.9	(1.2-2.5)	< 0.001
Valvular disease^2^	45 (3.0)	134 (8.9)	61 (4.1)	186 (12.4)	103 (6.9)	149 (10.0)	6	(5.1-6.8)	8.3	(7.2-9.4)	3.1	(1.6-4.6)	< 0.001
CEVD^1,2^	58 (3.9)	127 (8.5)	73 (4.9)	162 (10.8)	74 (4.9)	123 (8.2)	4.6	(3.6-5.6)	5.9	(4.9-7.0)	3.3	(2.0-4.5)	< 0.001
Hemiplegia or paraplegia^1,2^	17 (1.1)	88 (5.9)	31 (2.1)	101 (6.8)	36 (2.4)	90 (6.0)	4.7	(4.1-5.4)	4.7	(4.0-5.5)	3.6	(2.5-4.7)	< 0.001
Hypertension ^2^	230 (15.4)	437 (29.2)	311 (20.7)	443 (29.6)	366 (24.4)	457 (30.5)	13.8	(12.2-15.4)	8.9	(7.3-10.5)	6.1	(3.6-8.5)	< 0.001
CPD^1,2^	57 (3.8)	161 (10.8)	87 (5.8)	174 (11.6)	109 (7.3)	205 (13.6)	7	(6.0-7.9)	5.8	(4.8-6.8)	6.4	(4.9-7.9)	< 0.001
Diabetes													
- with complication^1,2^	23 (1.5)	42 (2.8)	20 (1.4)	33 (2.2)	25 (1.7)	53 (3.6)	1.4	(0.6-2.3)	1.5	(0.6-2.5)	0.8	(-0.9-2.5)	< 0.001
- without complication^1,2^	76 (5.1)	97 (6.5)	95 (6.3)	118 (7.8)	132 (8.8)	144 (9.6)	1.2	(0.7-1.8)	0.8	(0.4-1.3)	1.9	(1.5-2.3)	< 0.001
Hypothyroidism^2^	14 (0.9)	33 (2.2)	40 (2.6)	63 (4.2)	35 (2.3)	64 (4.3)	1.3	(0.8-1.7)	1.6	(1.0-2.2)	2	(1.0-3.0)	< 0.001
Peptic ulcer^1^	15 (1.0)	59 (3.9)	24 (1.6)	49 (3.3)	19 (1.3)	68 (4.5)	3	(2.2-3.7)	1.7	(1.1-2.2)	3.3	(2.3-4.2)	< 0.001
Peptic ulcer (ex. bleeding) ^2^	3 (0.2)	44 (2.9)	10 (0.6)	41 (2.7)	8 (0.6)	57 (3.8)	2.7	(2.1-3.3)	2.1	(1.6-2.6)	3.3	(2.4-4.1)	< 0.001
Liver disease^2^	36 (2.4)	89 (5.9)	40 (2.6)	93 (6.2)	46 (3.1)	101 (6.7)	3.5	(2.8-4.3)	3.6	(2.9-4.3)	3.7	(2.5-4.8)	< 0.001
- Mild liver disease^1^	32 (2.1)	49 (3.3)	34 (2.3)	41 (2.7)	38 (2.6)	52 (3.5)	1.2	(0.6-1.8	0.4	(-0.1-0.9)	0.9	(0.2-1.7)	< 0.001
- MSLD^1^	6 (0.4)	4 (0.3)	10 (0.7)	9 (0.6)	19 (1.2)	9 (0.6)	-0.1	(-0.3-0.1)	-0.1	(-0.3-0.1)	-0.6	(-1.1--0.2)	0.028
Any Tumor^1^	132 (8.8)	124 (8.2)	136 (9.1)	142 (9.5)	157 (10.4)	146 (2.2)	-0.5	(-1.6-0.5)	0.4	(-0.6-1.4)	-0.7	(-2.4-0.9)	0.627
Lymphoma^2^	18 (1.2)	29 (2.5)	26 (1.7)	30 (2.8)	16 (1.1)	25 (2.2)	1.3	(0.8-1.9)	0.6	(0.2-1.0)	1.1	(0.4-1.8)	< 0.001
Metastatic cancer^1,2^	40 (2.7)	120 (8.1)	75 (5.0)	119 (8.0)	71 (4.8)	119 (7.9)	-0.4	(-1.5-0.6)	0.6	(-0.4-1.7)	-1.7	(-3.2--0.1)	0.842
STWM^2^	125 (8.3)	117 (7.9)	140 (9.3)	148 (10.0)	153 (10.2)	128 (8.5)	5.4	(4.5-6.3)	3	(2.0-3.9)	3.2	(1.8-4.6)	< 0.001
Renal failure^1,2^	65 (4.3)	147 (9.8)	61 (4.1)	152 (10.1)	59 (4.0)	150 (10.0)	5.5	(4.6-6.4)	6	(5.2-6.8)	6	(5.1-6.9)	< 0.001
Rheumatic disease^1,2^	10 (0.7)	27 (1.8)	17 (1.1)	29 (1.9)	14 (0.9)	49 (3.2)	1.1	(0.7-1.6)	0.8	(0.3-1.3)	2.3	(1.7-2.9)	< 0.001
AIDS/HIV^1,2^	9 (0.6)	15 (1.0)	4 (0.2)	7 (0.5)	3 (0.2)	13 (0.9)	0.3	(0.0-0.7)	0.2	(0.1-0.4)	0.7	(0.4-0.9)	< 0.001
Blood loss anemia^2^	3 (0.2)	55 (3.7)	11 (0.7)	80 (5.4)	12 (0.8)	99 (6.6)	3.5	(3.1-4.0)	4.6	(4.2-5.0)	5.8	(5.2-6.3)	< 0.001
Deficiency anemia^2^	11 (0.8)	59 (3.9)	25 (1.7)	79 (5.3)	17 (1.2)	65 (4.3)	3.2	(2.5-3.9)	3.6	(2.9-4.2)	3.2	(2.1-4.2)	< 0.001
Coagulopathy^2^	17 (1.1)	45 (3.0)	18 (1.2)	64 (4.3)	30 (2.0)	95 (6.3)	1.9	(1.4-2.4)	3.1	(2.6-3.6)	4.3	(3.3-5.4)	< 0.001
FED^2^	20 (1.4)	106 (7.1)	32 (2.2)	123 (8.2)	59 (4.0)	152 (10.1)	5.7	(5.1-6.3)	6.1	(5.4-6.7)	6.2	(5.0-7.3)	< 0.001
Weight loss^2^	8 (0.5)	58 (3.9)	14 (0.9)	82 (5.5)	10 (0.7)	63 (4.2)	3.3	(2.8-3.8)	4.5	(3.9-5.1)	3.5	(2.9-4.1)	< 0.001
Obesity^2^	33 (2.2)	104 (7.0)	48 (3.2)	99 (6.6)	62 (4.1)	110 (7.3)	4.7	(4.1-5.4)	3.4	(2.6-4.2)	3.2	(1.9-4.5)	< 0.001
Alcohol abuse^2^	41 (2.7)	92 (6.1)	62 (4.2)	97 (6.5)	67 (4.5)	116 (7.7)	3.4	(2.7-4.1)	2.3	(1.6-3.1)	3.3	(2.1-4.5)	< 0.001
Drug abuse^2^	15 (1.0)	31 (2.1)	14 (1.0)	28 (1.8)	27 (1.8)	52 (3.4)	1	(0.6-1.4)	0.9	(0.6-1.2)	1.6	(1.0-2.3)	< 0.001
Dementia^1^	22 (1.5)	32 (2.2)	19 (1.3)	30 (2.0)	20 (1.3)	23 (1.6)	0.7	(0.1-1.2)	0.7	(0.3-1.2)	0.2	(-0.1-0.6)	< 0.001
Psychosis^2^	3 (0.2)	23 (1.6)	14 (1.0)	26 (1.7)	11 (0.7)	23 (1.5)	1.3	(0.9-1.7)	0.8	(0.4-1.2)	0.8	(0.3-1.3)	< 0.001
Depression^2^	56 (3.7)	121 (8.1)	83 (5.5)	140 (9.3)	92 (6.2)	154 (10.3)	4.4	(3.5-5.2)	3.8	(2.8-4.8)	4.1	(2.7-5.5)	< 0.001

Abbreviations: PCD = Pulmonary circulation disorders, PVD = Peripheral vascular disease, CEVD = Cerebrovascular disease, CPD = Chronic pulmonary disease, MSLD = Moderate and severe liver disease, STWM = Solid tumour without metastasis, FED = Fluid and electrolytic disorders.

* Sample size without weighted sampling fraction (i.e., observed sample size).

**Cochran *Q *test for heterogeneity of prevalence difference (Δ*_Chart-ICD_*) between chart review and administrative data across years.

^1 ^Charlson co-morbidities; ^2 ^Elixhauser co-morbidities.

^(a) ^Only recent myocardial infarction.

Indicators of accuracy of administrative data and of agreement between chart review data and administrative data by study year are presented in Table 2. For the 17 variables of the Charlson co-morbidity index, sensitivities ranged from 17.4% to 64.1% (median 36.5%) in 1999, from 22.2% to 64.6% (median 42.5%) in 2001 and from 8.4% to 75.6% (median 42.8%) in 2003. For the 29 Elixhauser co-morbidities, sensitivities ranged from 1.9% to 64.1% (median 34.2%) in 1999, from 10.5% to 66.5% (median 38.6%) in 2001 and from 5.1% to 76.5% (median 41.6%) in 2003. Out of 36 conditions, there was an increase in sensitivity for the 30 following conditions between 1999 and 2003: cardiac arrhythmias*, myocardial infarction, peripheral vascular disease, pulmonary circulation disorders, valvular disease*, cerebrovascular disease, hemiplegia or paraplegia, hypertension*, diabetes without complication, hypothyroidism, peptic ulcer, peptic ulcer excluding bleeding, liver disease, mild liver disease, moderate and severe liver disease, any tumour, metastatic cancer*, solid tumour without metastasis, blood loss anaemia, deficiency anaemia, fluid electrolytic disorder*, weight loss, obesity*, alcohol abuse, drug abuse, dementia, psychosis, depression (statistically significant for 6 of those(*)). A decrease was observed for the 6 following conditions: diabetes with complications, lymphoma, renal failure, rheumatic disease*, AIDS/HIV, coagulopathy (statistically significant for only 1 of them (*)).

**Table 2:** Agreement and Accuracy between Chart and Administrative Data by Study Year, N = 3'449*

	1999 (n = 1'158)				2001 (n = 1'195)				2003 (n = 1'146)				Comparison across years	
	
COMORBIDITIES	Se	PPV	Kappa		Se	PPV	Kappa		Se	PPV	Kappa		P-value**	
											
	*%*	*%*	k	(95% CI)	*%*	*%*	k	(95% CI)	*%*	*%*	k	(95% CI)	Se	PPV
Cardiac arrhythmias^2^	35.3	98.8	0.48	(0.38-0.54)	43.9	96.4	0.55	(0.48-0.62)	56.1	89	0.65	(0.57-0.70)^†††^	0.001	0.022
Congestive heart failure^1,2^	20.8	82.3	0.27	(0.17-0.32)	23.8	84.9	0.31	(0.22-0.37)	26.3	83.4	0.38	(0.28-0.44)	0.193	0.898
Myocardial infraction^1(a)^	43	56.9	0.47	(0.36-0.60)	71.5	61.4	0.65	(0.52-0.75)	56.2	69	0.6	(0.48-0.72)	0.109	615
PVD^1,2^	24.7	90.4	0.35	(0.22-0.41)	22.2	94.3	0.3	(0.20-0.34)	33.1	94.6	0.4	(0.30-0.47)	0.391	0.792
PCD^2^	34.1	82.9	0.47	(0.33-0.62)	36.1	79	0.5	(0.36-0.62)	38.6	78.5	0.51	(0.37-0.64)	0.934	0.984
Valvular disease^2^	31.3	93.8	0.44	(0.34-0.54)	30.8	93.7	0.43	(0.32-0.50)	60.6	88	0.65	(0.56-0.72)^††/†††^	0.001	0.816
CEVD^1,2^	35.7	78.1	0.46	(0.35-0.55)	31.7	71.2	0.43	(0.33-0.51)	47.3	78.6	0.54	(0.44-0.63)	0.18	0.951
Hemiplegia or araplegia^1,2^	17.4	90.6	0.31	(0.19-0.43)	26.1	96.2	0.39	(0.27-0.50)	37	94.3	0.45	(0.32-0.56)	0.307	0.86
Hypertension ^2^	49.6	94.1	0.6	(0.53-0.64)	66.5	94.9	0.71	(0.67-0.76)^†^	76.5	95.4	0.77	(0.73-0.81) ^††/†††^	< 0.001	0.921
CPD^1,2^	30.6	87.2	0.45	(0.35-0.53)	47.5	94.8	0.6	(0.52-0.67)^†^	44.2	82.8	0.54	(0.44-0.59)	0.052	0.097
Diabetes														
- with complication^1,2^	45.2	81.9	0.57	(0.42-0.72)	43	69.6	0.52	(0.35-0.68)	41.3	87	0.55	(0.42-0.68)	0.969	0.394
- without complication^1,2^	60	76.7	0.69	(0.59-0.76)	64.6	80.3	0.7	(0.62-0.77)	75.6	82.6	0.72	(0.64-0.78)	0.584	0.83
Hypothyroidism^2^	39	92.5	0.59	(0.40-0.74)	57.8	95.8	0.71	(0.59-0.80)	44.2	82	0.61	(0.47-0.72)	0.461	0.5
Peptic ulcer^1^	21.2	85.8	0.33	(0.19-0.49)	42	86	0.55	(0.38-0.67)	27.8	100	0.47	(0.32-0.60)	0.221	0.866
Peptic ulcer (ex. bleeding) ^2^	3.1	40	0.05	(0.00-0.23)	15.4	65.4	0.24	(0.10-0.41)	7.4	50.5	0.16	(0.05-0.33)	0.326	0.685
Liver disease^2^	36.3	90.3	0.5	(0.39-0.62)	35.8	84.4	0.48	(0.39-0.60)	44.8	97.8	0.63	(0.51-0.72)	0.329	0.265
- Mild liver disease^1^	49.4	81.4	0.6	(0.47-0.73)	48.4	68.3	0.56	(0.41-0.70)	51	78.5	0.66	(0.52-0.76)	0.916	0.299
- MSLD^1^	27.3	50	0.35	(0.08-0.78)	37.2	35.9	0.36	(0.14-0.63)	87.5	62.2	0.73	(0.46-0.90)	0.169	0.495
Any Tumor^1^	42.8	40.2	0.36	(0.28-0.46)	52.1	54.1	0.48	(0.39-0.55)	47.8	44.4	0.44	(0.35-0.52)	0.445	0.247
Lymphoma^1,2^	46.9	100	0.2	-	67.1	91.1	0.21	-	43.1	87.8	0.06	-	0.197	-
Metastatic cancer^1,2^	32	97.4	0.49	(0.38-0.59)	57.2	90.6	0.68	(0.59-0.75)	56.6	94.2	0.69	(0.61-0.74)^††^	0.007	0.502
STWM^2^	40.6	39.7	0.35	(0.26-0.44)	50	54.5	0.47	(0.38-0.54)	42.1	35.5	0.32	(0.25-0.42)	0.388	0.019
Renal failure^1,2^	42.7	97.6	0.61	(0.51-0.68)	38.2	94.8	0.52	(0.43-0.60)	36.6	92.8	0.49	(0.38-0.56)^†††^	0.204	0.351
Rheumatic disease^1,2^	37.2	100	0.54	-	55.4	93.8	0.69	-	26.8	92.2	0.41	-	0.049	-
AIDS/HIV^1,2^	64.1	100	0.78	(0.55-0.92)	50	100	0.67	(0.29-0.88)	16.8	66.7	0.27	(0.08-0.57)	0.086	-
Blood loss anemia^2^	1.9	39.7	0.03	(0.02-0.16)	10.5	74.9	0.17	(0.07-0.29)	5.1	40.8	0.08	(0.00-0.16)	0.163	0.477
Deficiency anemia^2^	12.7	66.2	0.2	(0.09-0.38)	20.1	67.2	0.29	(0.17-0.41)	13.4	50	0.22	(0.11-0.39)	0.691	0.623
Coagulopathy^2^	34.2	91.7	0.49	(0.34-0.65)	24.8	92.8	0.38	(0.24-0.51)	27.5	87.9	0.4	(0.28-0.52)	0.561	0.97
FED^2^	16.6	85.5	0.26	(0.15-0.37)	21.3	84.6	0.32	0.20-0.41)	32.9	86.7	0.43	(0.33-0.51)	0.031	0.978
Weight loss^2^	14.1	100	0.24	-	17.1	100	0.28	-	14.6	100	0.25		0.816	-
Obesity^2^	29.4	92.1	0.43	(0.34-0.55)	39.5	81.1	0.51	(0.42-0.63)	51.5	91.7	0.68	(0.57-0.76)^†††^	0.013	0.21
Alcohol abuse^2^	39	90.4	0.53	(0.43-0.65)	54.4	84.5	0.64	(0.55-0.74)	51.9	91.2	0.64	(0.55-0.73)	0.183	0.444
Drug abuse^2^	37.8	76.1	0.5	(0.32-0.67)	47.7	91.4	0.62	(0.45-0.79)	41.8	79.7	0.54	(0.38-0.65)	0.682	0.541
Dementia^1^	48.8	71.6	0.6	(0.42-0.74)	40.9	64.9	0.49	(0.31-0.66)	53.5	67	0.61	(0.42-0.75)	0.57	0.832
Psychosis^2^	10.4	70	0.18	(0.05-0.45)	41.2	74.1	0.52	(0.34-0.71)	42	90.5	0.57	(0.35-0.74)	0.112	0.647
Depression^2^	42.3	92.2	0.56	(0.48-0.67)	56.7	95.7	0.69	(0.60-0.76)	53.9	89.8	0.65	(0.54-0.70)	0.236	0.632

Se = Sensitivity, PPV = Positive predictive value, PCD = Pulmonary circulation disorders, PVD = Peripheral vascular disease, CEVD = Cerebrovascular disease, CPD = Chronic pulmonary disease, MSLD = Moderate and severe liver disease, STWM = Solid tumour without metastasis, FED = Fluid and electrolytic disorders.

^1^Charlson co-morbidities; ^2^Elixhauser co-morbidities.

^(a) ^Only recent myocardial infraction

*Sample size without weighted sampling fraction (i.e., observed sample size)

**Global Wald test

^† ^Difference was significant for comparison between 1999 and 2001, ^†† ^between 2001 and 2003, and ^††† ^between 1999 and 2003.

**Figure 1:**
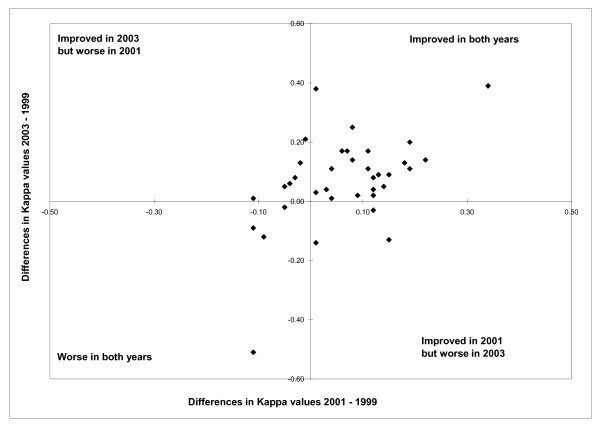
**Differences in Kappa Values between 1999, 2001 and 2003 of 36 Conditions Derived from Administrative Data Relative to Chart Review, N = 36**. Kappa value increased for 23 conditions (see the upper right quadrant) and decreased for 4 (see lower left quadrant) in 2001 and 2003 compared with 1999. Kappa value increased for 3 conditions in 2001 (see lower right quadrant) but decreased in 2003, and decreased for 6 conditions in 2001 but increased in 2003 (see upper left quadrant) compared with 1999.

Kappa values ranged from 0.05 to 0.78 in 1999, from 0.17 to 0.71 in 2001 and from 0.06 to 0.77 in 2003 across the 36 non redundant conditions from both Charlson and Elixhauser (see Table 2 and Figure 1). Between 1999 and 2003, Kappa values increased for 29 co-morbidities and decreased for seven (Table 2). The Figure 1 shows Kappa values comparing administrative data and chart data in 2001 and 2003 in relation to 1999 with more details. Of 36 conditions, the Kappa values increased for 23 conditions (see the upper right quadrant) and four decreased (see lower left quadrant) in 2001 and 2003 compared with 1999. Kappa values for three conditions increased in 2001 (see lower right quadrant) but decreased in 2003, and for six conditions decreased in 2001 but increased in 2003 (see upper left quadrant) compared with 1999. In most cases the changes in Kappa values across the years were not significant. For 2 co-morbidities the Kappa value increased significantly between 1999 and 2001, and for three co-morbidities the Kappa value increased significantly between 2001 and 2003 (for value increased in the 3 cases). In addition, for 4 co-morbidities the Kappa value increased significantly between 1999 and 2003.

In addition, weighted Kappa values between chart review and administrative data were 0.46 (95% CI: 0.44-0.51) in 1999, 0.52 (95% CI: 0.49-0.56) in 2001, and 0.54 (95% CI: 0.51-0.58) in 2003, when the Charlson index score was calculated using the weight developed by Charlson (using categorical variables with index scores of 0, 1, 2 or 3, and 4 or higher). These weighted Kappa values were 0.37 (95% CI: 0.34-0.40) in 1999, 0.45 (95% CI: 0.42-0.48) in 2001, and 0.48 (95% CI: 0.46-0.51) in 2003 when assessing the Elixhauser co-morbidity count (using categorical variables with co-morbidity counts of 0, 1 and 2, between 3 and 5, and 6 and higher).

## Discussion

Our study indicates that the accuracy of administrative data coded with ICD-10 improved slightly between 1999 and 2003. That improvement was evidenced by the increase of sensitivity for most co-morbidities across the five year period. However, we also found that, for some conditions (i.e. lymphoma, renal failure, rheumatic diseases, AIDS/HIV and coagulopathy), the accuracy of ICD-10 administrative data decreased somewhat over the period. These findings are of relevance to all jurisdictions interested in studying data quality trends as new coding systems are introduced.

There are several possible explanations for these results. First, professional coders were increasingly employed to code charts in Switzerland during the more recent years of the study period. However, there are few training programs for coders in Switzerland. Lay persons or clinically trained nurses and physicians are coding charts after only a short training period. Therefore, it is hard to avoid inter-coder variation in the quality of coding. Second, an APDRG reimbursement system was introduced in some hospitals. Therefore, this financial incentive may have triggered coders to code more conditions than previously. In our sample, the average number of diagnosis codes was 2.99 in 1999, 4.25 in 2001 and 4.91 in 2003. Third, coders' knowledge and skills in using ICD-10 coding methods and ICD-10 guidelines may have improved with time (i.e., a coding 'learning curve'), contributing to an improved adherence to coding guidelines. Fourth, administrative data quality improvement initiatives in Switzerland have been implemented with the creation of a coding unit at the Swiss Federal Statistical Office. This Office has developed and disseminated national coding rules for standardizing coding methods. Fifth, coders might have become more aware of the importance of certain conditions and paid more attention to coding these conditions. For example, we found that Kappa was 0.18 in 1999 and increased to 0.52 in 2001 and 0.57 in 2003 for obesity although this condition is not considered to be important for coding unless it directly contributes to the hospital stay. Sixth, it is possible that physicians documented clinical information better so that coders could translate the clinical information into electronic codes more easily. We also found that the accuracy of six conditions decreased over the study period. The accuracy of AIDS/HIV dropped dramatically. The sensitivity decreased from 64.1% in 1999 to 16.8% in 2003. While this drop appears substantial at first glance, we suspect that random error is a major contributor to this finding, as a result of the very small sample size used for judging the accuracy of this variable. In addition, PPV was rarely equal to 100%. There are several possible explanations for false positives: incomplete medical records used in this study; the research nurses could have missed some clinical diagnosis from chart review. Indeed, chart review is not a perfect reference standard.

Since the introduction of the ICD-10 coding system, only a few accuracy studies have been conducted. Our 2003 results for sensitivity and Kappa values were similar to those obtained in 2003 in a Canadian study [19]. Both studies employed the same methodology, including study designs, data collection process and definition of study variables. In Europe, Gibson and Bridgman [28] compared the accuracy of ICD-10 primary diagnosis coding in hospital administrative data versus charts. They studied a total of 298 general surgery records from the North Staffordshire Health Authority, United Kingdom, in 1996-1997. Coding errors occurred in 8% of records at the first character level, 9% at the second character level, 24% at the third character level, and 29% at the fourth character level. A recent study in Australia [29] demonstrated that the validity of ICD-10 administrative data was high in 2000 and 2001, two years after the introduction of the ICD-10 in that country, with sensitivities ranging from 0.58 to 0.97. In the future, more and more countries will be using ICD-10. The USA have plans to introduce ICD-10-CM in 2013 [30]. Use of administrative data and their validation will become increasingly important internationally in the future. The World Health Organisation (WHO) is continuously working on ICD-10 revisions and the production of ICD-11 is planned for 2015 [31]. Swiss administrative data have previously been studied, especially since the introduction of ICD-10 [17] in 1998. However, to date, only quality of coding reliability studies have been performed in the country [31,32], that have shown that about two thirds of the primary diagnosis codes had all five characters correctly coded in 1998 already [33] and that major improvements in the quality of coding occurred between 1998 and 2003 [34].

Administrative data are commonly used for trend analyses of conditions and quality of care assessment over time [19,28,29]. Such studies should be interpreted with caution. Indeed, we have observed in our study that the accuracy of several conditions improved or decreased over time. Such variations in accuracy over time could result in better or worse identification by time period. For example, the prevalence of AIDS/HIV decreased as a co-morbidity, apparently, from 0.6% in 1999 to 0.2% in 2003, when based on administrative data. However, the Figure 1 was stable, at 1.0% in 1999 and 0.9% in 2003, when based on chart review data. The decrease of the positive predictive value for AIDS/HIV from 100% in 1999 to 66.7% in 2003 could mislead quality of care studies focusing on this co-morbidity, as might occur if one wanted to study anti-HIV virus drug utilization, for example. In such studies, the utilization was likely to decrease over the years due to higher levels of misclassification of non-AIDS/HIV cases, with 33.3% in 2003 compared to 0% in 1999. Therefore, trend accuracy in the study period should, when possible, be considered in the interpretation of such studies.

Our study has several limitations. First, we used chart data as the reference standard to evaluate the validity of ICD-10 administrative data. Ideally, validity should be assessed whether the condition is truly present in a patient or not. In fact, this standard depends on the quality of medical charts. However, the extent of clinical information missing in the charts cannot be determined. Second, although our sample size was relatively large, it was limited in view of the low prevalence of most Charlson and Elixhauser co-morbidities in acute care patients. Actually, we chose to use similar sample sizes to those of our Canadian colleagues [19]. Thus, estimates of accuracy parameters for some rare conditions lacked precision and the observed changes of indicator values was significant in few cases only, but most significant changes corresponded to improvement with time. Third, the accuracy of administrative data may vary across hospitals [35], and from country to country. Therefore, generalizability of our findings to other jurisdictions is not certain and should be assessed through similar studies in other countries of coding accuracy over time. Fourth, the use of only one individual research nurse to abstract the data from each chart, and 2 individuals in total was another limitation. We examined inter-rater agreements and further trained the research nurses. Fifth, there were differences in the number of diagnosis codes recorded between the three hospitals. Moreover, the number of patients excluded from the non-teaching hospital was proportionately much larger than in the teaching hospitals. Thus, the type of hospital might influence our results. In addition, we used a convenience sample, not a representative sample of all hospitals in the country, the external validity of our results is thus limited. It is difficult to extend our specific results to other countries because coding rules are potentially different. For instance, the number of coded diagnoses varies between countries and could constitute a bias for international comparisons [36]. However, comparisons could be possible between countries using a selection of co-morbidities like Charlson co-morbidities [37]. Nevertheless, beyond the specific figure, the phenomenon is worth noting and is of relevance to all jurisdictions introducing new coding systems. Improved administrative data accuracy may relate to a coding 'learning curve' with the new coding system. In addition, as coding rules are being adapted constantly, e.g., in relation with DRG reimbursement schemes, one cannot take for granted that the measure of cormibidty is stable over the years.

However, our study has some strength. This is, to our knowledge, the first study assessing the evolution of accuracy of co-morbidity information derived from administrative data, measured at three time points over a five-year period shortly after the introduction of ICD-10. In an attempt to represent national hospital discharge data, we included both teaching and non-teaching hospitals because the validity of administrative data can vary by type of hospital [34]. Possible further investigations could include using the patient as the unit of analysis instead of each co-morbiditiy independently. This will constitute a different approach to the validity issue of administrative data.

## Conclusions

Our study demonstrates that the accuracy and reliability of co-morbidity information from ICD-10 administrative data improved slightly between 1999 and 2003. This improvement may be related to higher adherence to coding standards and systematic use of professional coders in Swiss hospitals over these years. This finding indicates that other countries should consider similar data accuracy assessments over time as new coding systems are introduced.

## Competing interests

The authors declare that they have no competing interests.

## Authors' contributions

JMJ carried out the data collection and performed the statistical analyses. JCL carried out the design of the study and coordination of data collection and participated in the analysis. HQ conceived and participated in the design of the study and statistical analysis. FB participated in the design of the study and facilitated the data collection. PT participated in the statistical analysis. WAG participated in the design of the study. BB participated in the study conception, design and coordination. All authors read and approved the final manuscript

## Pre-publication history

The pre-publication history for this paper can be accessed here:

http://www.biomedcentral.com/1472-6963/11/194/prepub
